# Infectious Diseases: A Clinical Approach, 2nd Edition

**DOI:** 10.3201/eid1206.060296

**Published:** 2006-06

**Authors:** David L. Paterson

**Affiliations:** * University of Pittsburgh, Pittsburgh, Pennsylvania, USA

**Keywords:** Book review, Australia, South PacificAllen Yung, Malcolm McDonald, Denis Spelman, Alan Street, Paul Johnson, Tania Sorrell, and Joe McCormack, editors

The recent award of the Nobel Prize to Barry Marshall and J. Robin Warren for their discovery of *Helicobacter pylori* and its role in gastritis and peptic ulcer disease has again put infectious diseases on to the front page of newspapers around the world. Notably, their initial work was performed in Perth, Australia. The clinical practice of infectious diseases, and associated research into microorganisms, has long been established in Australia. This book capitalizes on the expertise of infectious disease physicians and microbiologists in Australia and surrounding regions. Infectious Diseases: A Clinical Approach is written from an Australian perspective but has potential for a global audience.

The book’s first 2 parts deal with a general, clinical approach to the patient with a possible infection. This section is full of clinical "pearls," particularly relating to the patient with undifferentiated fever. Some sections, such as "Pitfalls in the Diagnosis of Serious Bacterial Infection, Especially Meningococcemia" or "Reasons Not To Treat Fever," are a joy to read and could be posted on the walls of emergency departments or general medical wards in every hospital. These sections are of relevance to internists and infectious disease fellows, but for a seasoned infectious diseases physician, they may not offer anything new.

Experienced infectious disease physicians, however, are likely to gain new insights into infections occurring in Asia, Australia, and the southwest Pacific region. A patient vacationing one day in Fiji or Tahiti may be visiting the emergency department of a hospital in North America or Europe the next day. While most physicians might search the Centers for Disease Control and Prevention website to find what infections are endemic in the region in question, or whether outbreaks have recently been reported there, this book provides context from authors working in the regions they describe. The clinical evaluation of infections in returned travelers and immigrants is also particularly well covered.

As an infectious disease physician working in the United States, I would not purchase this book to find detailed information on specific infections or specific antimicrobial agents. This is a book I would purchase for a first-year fellow or a resident who has enjoyed rounding with the infectious diseases service. The book is a gold mine of clinical advice based on obtaining a detailed history and performing a thorough physical examination. It is full of advice on "traps for young players" and ends with 28 golden rules of infectious disease practice. The book is dedicated to Richard Kemp, an infectious disease physician, who tragically died at the age of 50. Kemp was renowned as a clinician and would have undoubtedly endorsed the practical approach to infectious disease management advocated in this book.

